# P-1396. Compliance Rates to Updated CDC Guidelines and an Automatic Communication Intervention Following Positive Sexually Transmitted Infection Results in the Emergency Department

**DOI:** 10.1093/ofid/ofae631.1572

**Published:** 2025-01-29

**Authors:** Noor Taweh, Patrick Cahill, Jyoti Chhabra

**Affiliations:** University of Connecticut School of Medicine, Connecticut; Hartford Hospital, Hartford, Connecticut; Hartford Hospital, Hartford, Connecticut

## Abstract

**Background:**

The emergency department (ED) remains at the forefront of the growing sexually transmitted infection (STI) epidemic. Despite being a critical leverage point for STI management, current practices can be incongruent with recent 2021 CDC STI Guidelines. The transitory role of the ED poses challenges to establishing follow-up. We sought to assess the degree of deviation from CDC STI guidelines in the Hartford Hospital ED and optimize management by having positive STI tests automatically routed to the outpatient infectious disease (ID) clinic for follow-up.

**Methods:**

This was a retrospective, observational study of 342 patients from October 31^st^, 2021 to August 18^th^, 2023. Pre-intervention, ED providers were solely responsible for reviewing results and contacting patients to return for treatment. An intervention was initiated on October 31^st^, 2022, whereby positive STI test results from the ED were automatically rerouted to the ID clinic EPIC inbox, eliminating the modulatory role of ED providers. Manual chart review was conducted to evaluate compliance to CDC guidelines and parameters before and after the intervention, including the rate of referral to and follow-up with ID.

**Results:**

Significant deviations from CDC guidelines were observed: 78.8% of practitioners took inadequate sexual history, 95.0% did not order comprehensive STI screening (gonorrhea, chlamydia, HIV, and syphilis), and only 60.8% initiated empiric antibiotic therapy. Of those, only 71.6% were guideline-based therapies. This intervention was successful in decreasing the proportion of patients lost to follow-up; Prior to intervention, 3.9% of patients were seen in the ID clinic for follow-up which increased to 15.3% after intervention (p < 0.001).

**Conclusion:**

Although guidelines were updated almost 3 years ago, current practices remain incongruent with recommendations. Automatically rerouting positive STI results resulted in a higher rate of follow-up. We propose other hospital systems utilize a similar automatized system to minimize gaps in care and reduce burden on strained ED systems.

**Disclosures:**

**All Authors**: No reported disclosures

